# Diarrheal Diseases in Infancy*Written expressly for The Texas Medical Journal.

**Published:** 1915-08

**Authors:** A. Spencer Davis

**Affiliations:** Dallas, Texas


### Diarrheal Diseases in Infancy. *

BY DR. A. SPENCER DAVIS, DALLAS, TEXAS.

There is no disease of infancy which has received so much attention as diarrhea, and about which we knew so little until the last few years.

In text-books on children's diseases one sees the terms ileo colitis, entero colitis, cholera infantum, and summer complaint. Yet I feel sure that the clinical picture of these diseases is not clear to the minds of very many physicians, and after one has examined the child with diarrhea and has seen the stools, one is not sure whether he is dealing with a case of entero catarrh, ileo colitis or cholera infantum.

When one reviews the great amount of literature which has appeared in the medical journals of this country and abroad we find that all the descriptions of diarrhea there found can be brought under two types: (1) Infectious diarrhea. (2) Non-infectious. Only the first variety will be discussed in this paper. In order that there may be no confusion as to what is meant by infectious diarrhea, the characteristics will be described. When Finklesein made his discovery of the injuries produced by food, he was inclined to the opinion that all the diarrheas of infancy were produced by food of improper quality or of proper food in excess of the child's demands.

He was inclined to reject bacteria as being a cause for these diarrheas in early life. Recently he has greatly modified his view, and is now of the opinion that there is a type which we call infectious. This type of diarrhea has the following clinical picture with which I am sure nearly all physicians are familiar:

The outset resembles frequently the outset of typhoid fever and the things which make diagnosis positive are: (a) The early presence of blood and mucus in the stools. (b) A temperature which persists beyond twenty hours after all food is withheld.

The blood appears at first as streaks upon the mucus and as the disease progresses the blood increases in quantity. It is usually of a bright red color. When the inflammatory process reaches a marked degree, blood is replaced by pus. This is the clinical picture which one frequently sees in the dysentery of adults.

Early in the disease the child may not appear very sick and

Written expressly for The Texas Medical Journal.

the character of the stools may strongly contrast with the clinical condition of the patient. A number of years ago Dr. A. I. Kendall and his co-workers made an elaborate investigation of this type of diarrhea at the Boston Floating Hospital. Each summer they have renewed their investigations and rendered a report of their work. They were astonished to find that this particular type of diarrhea is always produced by one of the following types of bacteria: (1) Dysentery bacillus either of Shiga or Flexner type. (2) Colon bacillus. (3) A streptococcus. (4) Gas bacillus. The colon bacillus is an organism which is capable of changing its characteristics. It may be either a protein decomposer or a carbohydrate fermenter. The products of protein decomposition are very toxic, while those derived from fermentation of carbohydrates are relatively non-toxic.

When the colon bacillus produces this particular type of diarrhea it has become a protein decomposer. Having isolated these organisms from the stools of children, a series of experiments were undertaken to determine upon what sort of culture medium they grow best, and to see if it would be possible to influence their growth in the intestinal tract by changing the diet. It was found that the addition of milk sugar to the culture medium caused a marked inhibition of the growth of all these bacteria with the exception of the gas bacillus, which instead of being inhibited by the milk sugar, was greatly accelerated in its growth.

They attempted to use a 5 per cent solution by mouth with beneficial results. Since some of the cases will have an infection with gas bacillus and as these are made worse by the administration of milk sugar solution it is important to know when this type of organisms is the cause of the diarrhea. A method to determine this, which is very simple, is to add a small particle of the stool to some sweet milk in a test tube, the mixture is heated to 80 degrees centigrade for a few minutes, and the tube is kept in a warm place twenty-four hours. All the bacteria are destroyed, except the gas bacillus, and when it is present there is noted the following day a stormy fermentation in the test tube with large bubbles of gas.

*Treatment.*—To get rid of the blood and mucus is one of the first indications for treatment. To accomplish this end we have found one drug particularly effective, that drug is magnesium sulphate. But in order to accomplish the purpose for which it is used, it must be given in a certain manner. For young infants we use a teaspoonful of a saturated solution every thirty minutes, until the stools are free from blood and mucus, which usually happens within the first twenty-four hours. A single large dose of magnesium sulphate will not accomplish the purpose, but when the dose is frequently repeated as described above, we soon have the intestinal tract containing a solution of magnesium sulphate throughout its extent.

This hyper-tonic solution causes a marked exudation from the blood stream, which, as it passes from the blood into the intestines washes out the crypts and folds of the mucous membrane. Although the magnesium sulphate solution increases the number of stools, the tenesmus is relieved, and although the bowels may move a great many times from the purgative, no depression of the patient is produced.

As soon as the desired action is attained from the magnesium sulphate the administration of food is begun. If the gas bacillus is not present it is advisable to begin feeding with a 5 per cent milk sugar solution, and slowly add diluted skim milk, increasing the strength of the food until the child is on a full diet.

If the child is upon the breast it is given small feedings at first, gradually increased to full feedings. If the gas bacillus is present, as a causative factor, a food must be given which is free as possible from carbohydrates. Of all the foods tried buttermilk is the most efficient, which is diluted and given according to the age of the child.

Frequently the pathology of this type of diarrhea is confined entirely to the rectum and sigmoid. When this is the case irrigations are beneficial, but it is doubtful if they are of marked assistance when the process is high up in the colon. Once a day is often enough to irrigate. Nearly every one has some particular solution for irrigation which he thinks particularly effective, but perhaps it is more the mechanical cleansing of the mucous membrane by water than the antiseptic action of the substance used.

Cases which are of long standing are more difficult to manage, and here we have used Argyrol solution. When astringent action is needed we have used a 5 per cent solution of nitrate of silver after a preliminary cleansing of the bowels with plain boiled water. This injection is followed immediately by normal saline to stop the action of the silver.

Following the measures outlined above, the number of stools will frequently be reduced to four or five a day, but will continue to be diarrheal in character. It is in this situation that bismuth renders us marked service and is best given in large doses.

The use of calomel is only mentioned to be condemned and one will frequently observe that a simple diarrhea will be made worse and blood and mucus made to appear in the stool after its use.

---

### A Few Surgical Don'ts.

Don't irrigate deep wounds; it only macerates the tissues and retards granulation.

Don't fail to have an X-ray taken of all fractures. This is particularly important in cities.

Don't operate on your patients as soon as they will submit; better lose to another doctor through delay than to an undertaken through haste.

Don't operate just to remove or repair something. The end result is what the patient is looking for.

Don't constantly change the dressings on clean wounds. "It will never get well if you pick it."

Don't forget that an irregular show with scant flow may mean ectopic pregnancy.

Don't overlook retention in cases of frequent micturition after operation. Catheterize to find out.

Don't withhold the stomach pump in prolonged post-operation vomiting. Prompt lavage hurts no one, and may save many.

Don't drag on the mesentery. It carries the nerve and blood supply to the viscera.

Don't eviscerate your patient to find the field of operation. If you can't do better than that, stay out.

Don't forget the prostate in obscure conditions; much trouble often lurks there.

Don't bluff yourself that a hospital in your town warrants you to do things you know you have not been trained to do. There are many operators, but few surgeons. If you can't be the latter, don't try to be the former.

Don't think that a tile floor, white suit, and rubber gloves will change your spots.

Don't fool yourself into the belief that four walls hide the sins of surgical omission and commission.

Don't drain gall-bladders as a routine. Many should be removed.

Don't overlook an opportunity to improve your judgment of gross pathology. Mr. Internist, please take note.

Don't rely on pain temperature and pulse in guessing the severity of appendicitis. They are false gods. Eighty per cent polymorphonuclear leucocytosis means pus or gangrene.

Don't sew the skin so tightly—mere approximation is all that is needed.—*Chas. H. Venable, M. D.*

---

THE WORST.—"Germany, with France on one side of her and Russia on the other, with France drawing her this way and Russia drawing her that, is in as bad a pickle as Artemus Ward's invalid."

The speaker was General Nathan Bedford Forrest of Memphis. He continued:

"To Artemus Ward, you know, a man once said:

" 'I've got a toothache and earache. Could anything be worse?'

" 'Oh, yes,' said Artemus. 'I know a chronic sufferer of eighteen years' standing. He's worse. For his complaint is inflammatory rheumatism coupled with St. Vitus' dance.' "—*New York Tribune.*

---

### A Hoosier Hymn of Peace.

The following lines were written by George B. Lockwood, for a recent issue of the *Muncie Press*, and they express somewhat of the sentiment which Americans entertain at the present moment:

"I would rather be a Hoosier far removed from war's alarm, eating roasting ears and chicken from an Indiana farm, than to be a German warrior on a Belgium field of gore with a bullet in my gizzard and my plate held up for more. I would rather be a Hoosier, working at my prosy job, than to be where Russian bullets with my innards might play hob. I would rather dig potatoes in my little tuber patch than to be a human target in a daily shooting match. I would rather read war extras seated in my humble cot than to have my name and address in the list of brave men shot. For the war-lords at a distance, war is surely something great, as their armies mow down thousands in a fight o'er real estate. What though mothers, wives and orphans weep an ocean full of tears, what though fire, sword and famine with their ruin fill the years—be he czar or king or kaiser, he as dearly loves the tramp of his armies in commotion, as his phiz upon a stamp. They may sound in song and story praise of death in battle's roar, but what's the use of glory when there's crepe upon your door? I would rather plant a rose bush than to kill my fellow man, even though the work's conducted on a broad and wholesale plan, and I'm glad I'm not a subject of the powers at war today—it is good to be a Hoosier and to live so far away."

